# *EDAR *mutation in autosomal dominant hypohidrotic ectodermal dysplasia in two Swedish families

**DOI:** 10.1186/1471-2350-7-80

**Published:** 2006-11-24

**Authors:** Lisbet K Lind, Christina Stecksén-Blicks, Kristina Lejon, Marcus Schmitt-Egenolf

**Affiliations:** 1 Department of Medical Biosciences, Medical and Clinical Genetics, Umeå University, SE-901 85 Umeå, Sweden; 2 Department of Odontology, Pediatric Dentistry, Umeå University, SE-901 85 Umeå, Sweden; 3 Department of Public Health and Clinical Medicine, Dermatology and Venereology, Umeå University, SE-901 85 Umeå, Sweden

## Abstract

**Background:**

Hypohidrotic ectodermal dysplasia (HED) is a genetic disorder characterized by defective development of teeth, hair, nails and eccrine sweat glands. Both autosomal dominant and autosomal recessive forms of HED have previously been linked to mutations in the ectodysplasin 1 anhidrotic receptor (EDAR) protein that plays an important role during embryogenesis.

**Methods:**

The coding DNA sequence of the *EDAR *gene was analyzed in two large Swedish three-generational families with autosomal dominant HED.

**Results:**

A non-sense C to T mutation in exon 12 was identified in both families. This disease-specific mutation changes an arginine amino acid in position 358 of the EDAR protein into a stop codon (p.Arg358X), thereby truncating the protein. In addition to the causative mutation two polymorphisms, not associated with the HED disorder, were also found in the *EDAR *gene.

**Conclusion:**

The finding of the p.Arg358X mutation in the Swedish families is the first corroboration of a previously described observation in an American family. Thus, our study strengthens the role of this particular mutation in the aetiology of autosomal dominant HED and confirms the importance of EDAR for the development of HED.

## Background

Hypohidrotic ectodermal dysplasia (HED) is a congenital disorder characterized by abnormal morphogenesis of structures of ectodermal origin that may result in varying degrees of ectodermal defects such as hypodontia or anodontia, hypotrichiosis or alopecia, hypohidrosis or anhidrosis as well as nail abnormalities [1]. Conical teeth are a telltale sign in the HED patients, however other symptoms may vary between different individuals, even within the same family. The reduced or absent ability to regulate body temperature by sweating may lead to life-threatening hyperthermia, especially in very young children with an undiagnosed disorder.

The most prevalent form of HED is inherited as an X-linked condition [2] but autosomal dominant [3] and autosomal recessive [4] forms of the disorder are also found, albeit at a much lower frequency. According to the literature the most distinguishing feature for the HED variants appears to be the respective inheritance pattern [5].

HED may result from defects in any of three interacting proteins; ectodysplasin, EDAR or EDARADD. Ectodysplasin [6] is a soluble ligand protein that interacts with the transmembrane receptor EDAR, ectodysplasin 1 anhidrotic receptor [7-10] that is a death domain containing member of the tumor-necrosis factor receptor family. The intracellular part of EDAR subsequently binds to the adapter protein EDARADD (EDAR-associated death domain [11]) that links the receptor to downstream NF-κB signaling pathways [12].

Mutations in the gene coding for ectodysplasin, EDA situated on chromosome Xq12-q13.1, were the first to be found linked to HED [13]. The two most common splice isoforms of EDA (EDA-A1 and EDA-A2) bind the two distinct receptors EDAR and XEDAR respectively. They display different functions, where EDA-A1 appears to be essential for several skin appendages, and EDA-A2 probably is responsible for developmental timing and completion [14]. Later, both autosomal dominant and autosomal recessive variants of HED were found to be caused by different mutations in the EDAR gene on chromosome 2q11-q13 [15]. Autosomal recessive HED was also found to result from a mutation in the EDARADD gene on chromosome 1q42.2-q43 [16]. Interestingly, HED caused by mutations in either EDA or EDAR has been detected in several other species, including cattle [17], dogs [18] and mice [9,19,20]. Here we report the identification of a non-sense mutation in the EDAR gene in two large Swedish families with autosomal dominant (AD) HED.

## Methods

### Family material

**Figure 1:**
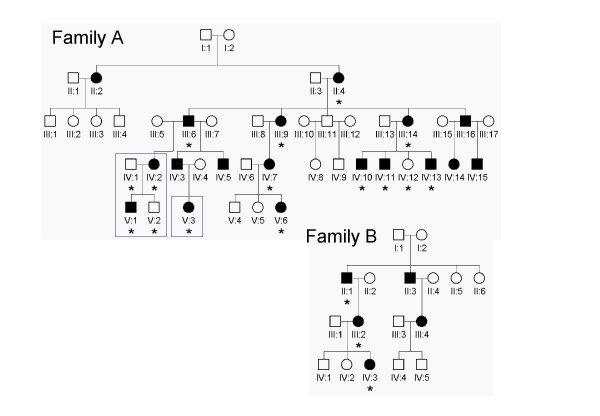
**Pedigrees of two Swedish families with autosomal dominant hypohidrotic ectodermal dysplasia**. Round symbols denotes women; square symbols, men; filled symbols, individuals affected by AD HED; * individuals subjected to DNA sequencing. The nuclear family chosen for the initial screening of *EDAR *and the girl shown in figure 2 are indicated by boxes.

Two families from the same geographical region in northern Sweden were investigated in this study. Both families included several members who suffered from various symptoms of AD HED (Fig. 1). Approval from the local medical ethics committee was obtained prior to the onset of the study and genetic material was obtained from the subjects after informed consent was obtained. Genomic DNA was prepared from buccal swabs from 18 individuals (15 AD HED patients and three unaffected relatives) according to standard protocols.

### Clinical description

**Figure 2:**
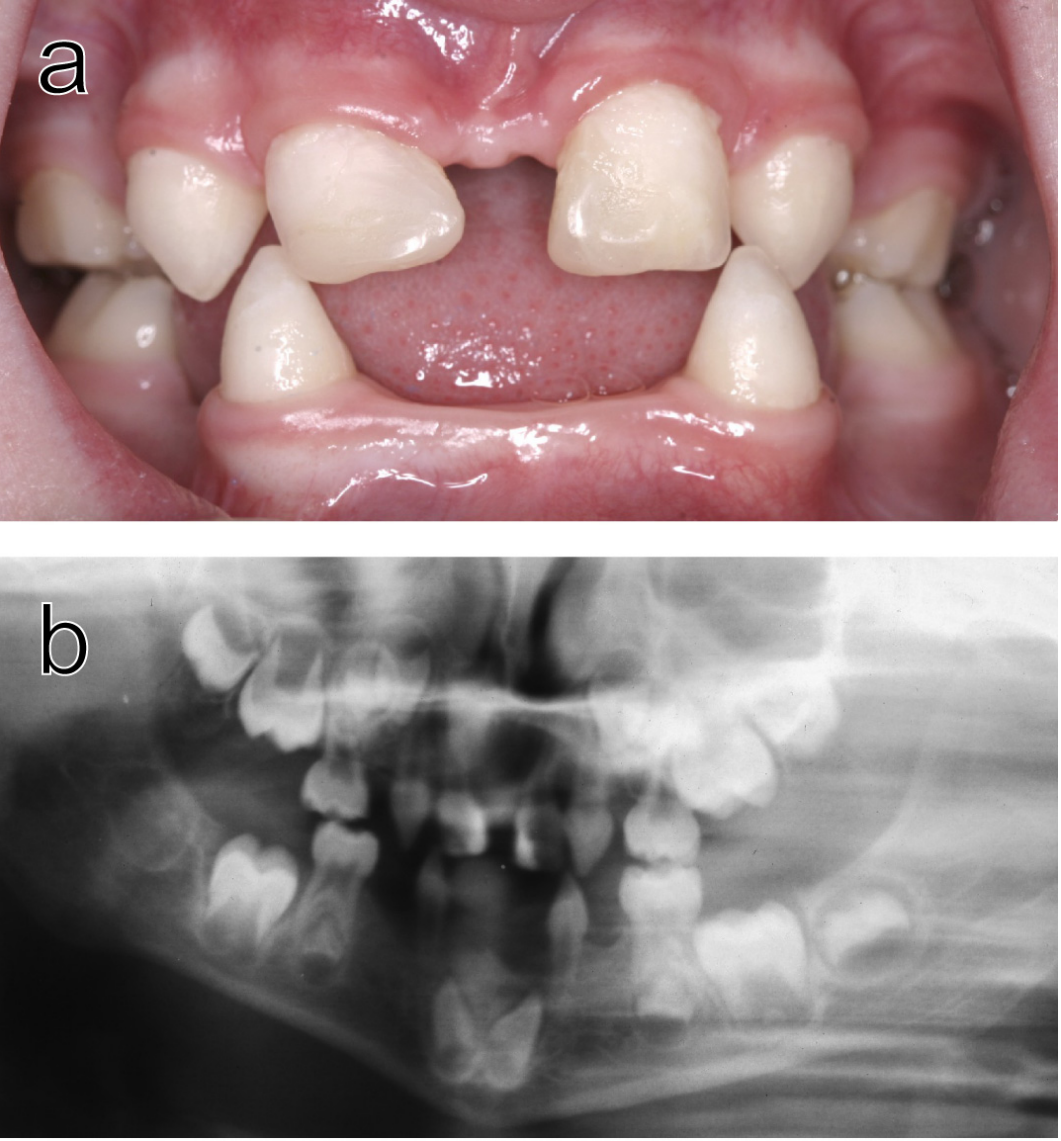
**Dental abnormalities in a 5-year-old girl from family A**. a) Intraoral view. Note that the upper incisors have been restored with composite material to disguise their original conical shape. b) Ortopantomogram showing absence of ten primary and 11 permanent teeth in the jaws of the same individual.

The phenotype of the AD HED patients included hypodontia with varying number of primary and permanent teeth absent (Fig. 2), hypohidrosis, and mild hypotrichiosis in accordance with an earlier description of AD HED [21,22]. The affected individuals from the Swedish families lacked 3 to 16 permanent teeth and a common sign observed in all affected persons was the absence of 1 to 4 permanent mandibular incisors. In addition to the dental anomalies the affected family members showed varying, but general mild, symptoms from skin and hair.

### PCR amplification of genomic DNA, DHPLC analysis and mutation detection

The *EDAR *gene was screened for mutations by denaturing high performance liquid chromatography (DHPLC) [23] of PCR products from eleven *EDAR *exons with flanking intron sequences (Table 1). Ten intronic oligonucleotide primer pairs for the *EDAR *gene were designed from the genomic sequence NT_022171 [24]. PCR products were amplified from a nuclear AD HED family of four individuals (Family A individuals IV:1, IV:2, V:1 and V:2) as well as one more distantly related AD HED patient and two unrelated healthy individuals. Initially temperature gradient PCR was used to determine the optimal annealing temperature for each primer pair, after which all individual samples were amplified by PCR and subjected to heteroduplex formation, in which the PCR products were slowly cooled from 95°C to room temperature, at 1.5°C/min. During all thermal reactions a PTC-200 Peltier Thermal Gradient Cycler (MJ Research, Waltham, MA, U.S.A.) was used.

**Table 1:** Primers used in the analysis of the *EDAR *gene. Intronic primer sequences, annealing temperatures and sizes of PCR products suitable for DHPLC analysis and DNA sequencing of the *EDAR *gene.

Exon no.	Forward primer (5'-3')	Reverse primer (5'-3')	Optimal annealing temp.* (°C)	Fragment size (bp)
2	TTTGCTGGAAGGCACCTTAT	AGAGGCCAAGAAACAGTCCA	58–62	243
3	ACCCCCTTCCTATGTCAACC	CAGGCTCAGGGCAACAAT	56–62	292
4	CGGCAAGAGTAGCTTCTGGA	GCAGTATCCATGACCCCTGT	51–63	397
5	GTGCTCTCTGCACCAGTCC	GACCGGCTCTTTCCTACACC	52–63	246
6	AGCTCTGTGGCAGCGTCT	CCTCTCCTCTTCTGAGCTTTCA	51–62	228
7 + 8	GGAGTCCTGGAGGGAAGACC	AGCATGTGAGAGCAGAAGCA	60	468
9	AGAGCAGGGTTGGGCTGAG	GCTAGCCTGTCAGTTCACTCG	51–63	248
10	AGGTGCCCAGTAAACACCTG	CGTCTTGCAGGAGAGCTGAT	51–63	400
11	CCTGCTGACATGGAGGATTT	CTCAGTTCCCCTCACAGGAG	51–63	234
12	GACCTTCTATTGACTGTGACTTGC	CAGTCTTTTGGCACCACTCA	51–63	461

*As indicated by gradient PCR reactions.

The Wavemaker™ software (Transgenomic, San Jose, CA, USA) was used to predict the optimal temperature for heteroduplex separation and acetonitrile gradients. Heteroduplexes were resolved from the corresponding homoduplexes using the WAVE system (Transgenomic, San Jose, CA, USA), an automated HPLC with a DNA separation column.

Exon fragments that showed a suspected sequence variation co-segregating with the disease were bi-directionally sequenced using Big Dye termination kit V3.0 (Amersham Biosciences) and the ABI 3730 DNA analyzer (Applied Biosystem). The same oligonucleotide primers that were used for PCR amplification were also used in sequencing reactions. Alignments and sequence comparisons were carried out using the BioEdit 5.0.7 software [25].

## Results

**Figure 3:**
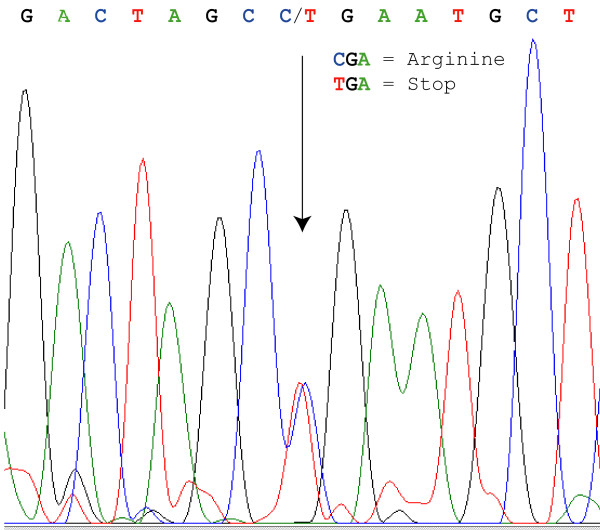
**DHPLC and sequence variance in affected and non-affected individuals**. DNA sequence from exon 12 of the *EDAR *gene in an AD HED patient. An arrow indicates the disease associated C/T mutation in sequence position c.1072.

DHPLC analysis of amplified PCR products from the 11 coding EDAR exons obtained from affected and unaffected family members indicated potential peak pattern variations in some exons but it was only one prominent variation that co-segregated with the disease phenotype. This was a conspicuous alteration in the peak pattern for exon 12, most noticeable at a denaturation temperature of 63°C. PCR fragments from exon 12 were subsequently bi-directionally sequenced from affected and non-affected individuals. Sequencing revealed a C to T transition at nucleotide position c.1072 of the *EDAR *gene (Fig. 3). Sequence positions are according to the DNA sequence obtained from the *EDAR *mRNA sequence with the ATG adenine as position +1 [26]. The mutation changes a CGA arginine codon to a TGA stop codon and is present in all affected, but no unaffected individual. In addition to the terminating mutation, sequencing also revealed two polymorphisms not related to the disease symptoms, a silent change at position c.1056 of a T to C in the third position of a TGC cysteine codon, and a T to G exchange at position c.1389 in the 3' untranslated region 42 nucleotides downstream of the native stop codon in exon 12.

## Discussion

In this report we have studied two presumed unrelated Swedish AD HED families stemming from the same small geographical region, and have identified an identical C to T mutation in exon 12 of the *EDAR *gene present in patients from both families. The mutation changes an arginine CGA codon into a TGA nonsense codon, thus introducing a truncation of the cytoplasmic portion of the EDAR protein at amino acid position 358. Thereby the p.Arg358X mutation leads to complete abolishment of the death domain region, amino acid 367 to amino acid 431, of the EDAR protein.

Two additional genetic variants were also detected in the PCR fragment that contained exon 12 of the *EDAR *gene; one polymorphism was a silent change, a T to C, just 15 nucleotides upstream of the p.Arg358X mutation, the other polymorphism, a T to G change, was located in the untranslated region downstream of the native stop codon of the *EDAR *gene. None of these polymorphisms appear to be associated with the AD HED disease, since the first variant was found both in a few healthy and in affected individuals and the second variant was only detected in two related healthy individuals, a parent and child. The most frequent sequence variant in both polymorphic loci, T, was found in homozygotic form at position c.1056 and position c.1389 in both healthy and affected individuals. Interestingly, the first polymorphism, c.1056 T to C was also detected in a previous study [15], but in that material the C allele was predominant and not the T allele as in our study. Likewise, in the published EDAR sequence [26] the nucleotide at position c.1389 is given as G, whereas in our material the G allele was only present as one copy each in two related individuals and all other individuals were homozygous for the T allele.

No known relationship exists between the two Swedish AD HED families, but since both families carry the same p.Arg358X mutation it is tempting to speculate that there is a previously unidentified relationship, especially when considering that the families originate from the same restricted geographical area.

All individuals affected by AD HED demonstrated absence of permanent teeth, albeit the actual number of missing teeth differed between the patients. Apart from the obligate odontological findings, other ectodermal symptoms were non-specific and, if present, generally mild. This is in contrast to a recently described family with AD HED where the affected individuals appear to have more pronounced symptoms overall [27]. Even though the actual mutation has not been identified in that family the disease gene maps to the same chromosomal region as the *EDAR *gene and it is very plausible that a mutation in the *EDAR *gene is the culprit in that AD HED family also.

The variable degree of expression of symptoms in individuals with AD HED is interesting and can probably be explained to some extent by different causative mutations. However, the variability of symptoms in teeth and other organs in individuals carrying the same *EDAR *mutation is intriguing and invites speculation to the cause. At least three different scenarios can be envisioned, two directly related to the nature of the *EDAR *mutation and one involving other gene products. The dominant negative action of the p.Arg358X mutation in AD HED could result from prevention of proper trimerization of the TNFR-like EDAR protein due to the lack of an intact death domain. Functional EDAR trimer complexes are required for accurate interactions with EDARADD and subsequent intracellular signaling [16]. Since EDAR trimers in AD HED patients most likely are formed by random combinations of normal and mutant variants of the protein, the proportion of functional EDAR homo-trimers in different individuals during embryogenesis and later developmental stages could vary. This variation could lead to a more or less severe clinical outcome due to chance deviations in the level of proper cell signaling in the ectoderm. An alternative explanation for the dominant-negative effect of the p.Arg358X mutation might be that the mutation leads to an altered subcellular location of the protein as compared to the non-mutated protein in analogy to what has been observed in studies of the orthologous mutated and wild-type Edar protein in mouse cell cultures [28]. A third scenario might be dissimilarities in the interactions between the mutant EDAR protein and different non-pathogenic variants of the ectodysplasin and EDARADD proteins.

So far, a limited number of *EDAR *mutations have been reported; five dominant mutations [15,29], six homozygous recessive mutations [15,29,30], and five compound heterozygous recessive cases [15,29,31], (Table 2). Interestingly, the mutation detected in the Swedish families is the same as one of the first autosomal dominant mutations described [15]. One of the two previously studied American families with autosomal dominant HED [15] was first described in 1987 [22] but it is unclear which mutation is carried in this family. The reason for identical mutations found in families on two different continents is not known. Perhaps there is a preference for mutations of the C in position c.1072 or it might even be that the Swedish and American families are related since many residents of Sweden migrated to North America during previous centuries.

**Table 2:** HED mutations in the *EDAR *gene

Inheritance	Genotype	Location	Sequence change*	Protein change	Predicted effect	Reference
AD	heterozygous	exon 12	c.1072C→T	Arg358Ter	truncated protein, no DD	present study, [15]
			c.1129C→T	Leu377Phe	altered DD	[29]
			c.1237A→C	Thr413Pro		[29]
			c.1253T→C	Ile418Thr		° [29]
			1259G→A	Arg420Gln		[15] [29]
AR	homozygous	intron 2	ΔIVS2 -25 to -8	disturbed splicing of exon 3		[15]
		exon 4	c.259T→C	Cys87Arg	altered LBD	[15]
		exon 8	c.718ΔAAAG	frame shift	truncated protein, no trimers formed	[30]
		exon 12	c.1144G→A	Gly382Ser	altered DD	[30]
		exon 12	c.1208C→T	Thr403Met	altered DD	[29]
		exon 12	c.1302G→T	Trp434Cys	altered DD	[29]
	compound heterozygous	intron 2	IVS2 +1G→A	exon 3 skipping	no stable transcript	[31]
		exon 12	c.1124G→A	Arg375His	altered DD	
		exon 3	c.140G→A	Cys47Tyr	altered LBD	[29]
		intron 6	IVS6+1G→A	disturbed splicing of exon 6		
		exon 4	c.266G→A	Arg89His	altered LBD	[15]
		at least exon 4	Δ ≥ exon 4	?	no protein?	
		exon 4	c.266G→T	Arg89His	altered LBD	[29]
		intron 6	IVS6+1G→A	disturbed splicing of exon 6		
		exon 4	c.329A→C	Asp110Ala	altered LBD	[29]
		exon 5	c.442T→C	Cys148Arg	altered LBD	

HED, hypohidrotic ectodermal dysplasia; EDAR, ectodysplasin 1 anhidrotic receptor; AD, autosomal dominant; AR, autosomal recessive; ° presumed AD; DD, death domain; LBD, ligand binding domain; Δ, deletion; IVS, intervening sequence.

* Sequence positions according to the *EDAR *cDNA sequence with the initiator adenine as position +1 [26].

## Conclusion

This is the first study corroborating the p.Arg358X mutation in the *EDAR *gene and it supports the notion that EDAR is one of the key proteins in the development of HED. Thus, our report strengthens the role of this particular mutation in the etiology of autosomal dominant HED and confirms the importance of EDAR in the development of HED.

## Competing interests

The author(s) declare that they have no competing interests.

## Authors' contributions

LKL made substantial contributions to the conception of the project, designed the molecular genetic studies, carried out and analyzed the experiments, interpreted the results, drafted and revised the manuscript.

KL participated in the interpretation of data, and helped to draft and revise the manuscript.

CSB made substantial contributions to conception and design, acquisition of data and coordination and helped to draft the manuscript.

MSE conceived and coordinated the study and helped to draft the manuscript.

All authors read and approved the final manuscript.

## Pre-publication history

The pre-publication history for this paper can be accessed here:


